# The role of active constituents of in traditional Chinese medicine for primary osteoporosis: a mechanistic review

**DOI:** 10.3389/fendo.2025.1647984

**Published:** 2025-09-16

**Authors:** Chaoqun Song, Lingfeng Zeng, Changwei Zhao

**Affiliations:** ^1^ Jiangxi University of Chinese Medicine, Nanchang, Jiangxi, China; ^2^ The Second Clinical Medical College of Guangzhou University of Chinese Medicine, Guangzhou, Guangdong, China; ^3^ Changchun University of Chinese Medicine, Changchun, Jilin, China; ^4^ The Affiliated Hospital of Changchun University of Chinese Medicine, Changchun, Jilin, China

**Keywords:** primary osteoporosis, traditional Chinese medicine, active constituents, mechanism, signaling pathways

## Abstract

Primary osteoporosis (POP) is a systemic metabolic bone disorder marked by diminished bone density and deterioration of bone microstructure, presenting a considerable challenge to global public health due to its widespread occurrence and heightened fracture risk. Although conventional western pharmaceutical treatments are efficacious, they are often associated with adverse events. Conversely, traditional Chinese medicine (TCM) exhibits distinct potential owing to its multi-targeted and multi-pathway regulatory benefits. This systematic review elucidates the molecular mechanisms of flavonoids, polyphenols, saponins, polysaccharides, coumarins, and alkaloids in the prevention and treatment of POP. The study elucidates the mechanisms of action by modulating critical signaling pathways, including the Wnt/β-catenin, RANKL/OPG pathways and so on, thereby facilitating osteoblast differentiation, suppressing osteoclast activity, and ameliorating oxidative stress, inflammation, and dysbiosis of the intestinal microbiota, ultimately restoring the balance of the bone microenvironment. This research aims to advance the development of innovative POP medications based on TCM principles and to provide scientific validation for individualized therapy.

## 1 Introduction

Primary osteoporosis (POP) is a systemic metabolic bone disorder characterized by diminished bone mass, deterioration of bone microarchitecture, and heightened bone fragility. It is predominantly observed in postmenopausal women and older males. Clinically designated as the “silent killer”, hip fracture resulting from osteoporosis (OP) frequently represent “the final fractures an individual endure in their lifetime”. Globally, one-third of women and one-fifth of men over the age of 50 will experience a fracture attributable to OP (1). A 2021 meta-analysis found that the global prevalence of OP is 18.3%, with women accounting for 23.1% and men for 11.7% in the general population across various pooled sample sizes (2). The causes are multifaceted, encompassing estrogen insufficiency, aging, and additional variables (3). The treatments are varied, encompassing contemporary pharmacological medicines, traditional Chinese medicine (TCM), physical therapies, and additional modalities.

Current pharmacological interventions primarily consist of bisphosphonates (4), receptor activator of nuclear factor kappa-B ligand(RANKL) inhibitors (5), sclerostin inhibitors (6), and active vitamin D, along with its analogs (7). TCM predominantly encompasses herbal medicine, acupuncture, and various other comprehensive intervention measures (8–10). Moreover, consistent physical activity has been shown to influence the management of OP positively (11). Despite the beneficial effects of these methods on the treatment of POP, certain limitations persist. For instance, although bisphosphonates commonly used in clinical can inhibit bone resorption, there are adverse reactions such as osteonecrosis, atypical femoral fractures, and esophageal cancer (12). Teriparatide can enhance osteoblast activity, thereby stimulating bone production, however, it may result in undesirable effects, including limb pain, muscle spasms, fractures, and elevated calcium levels (13). Although, Chinese herbal formula exhibits multi-target synergistic benefits, their complicated ingredients frequently result in an ambiguous pharmacodynamic material foundation, and the isolation and study of single compounds may inadequately reveal their holistic therapeutic effects and underlying mechanisms of action (14, 15).

**Figure 1:**
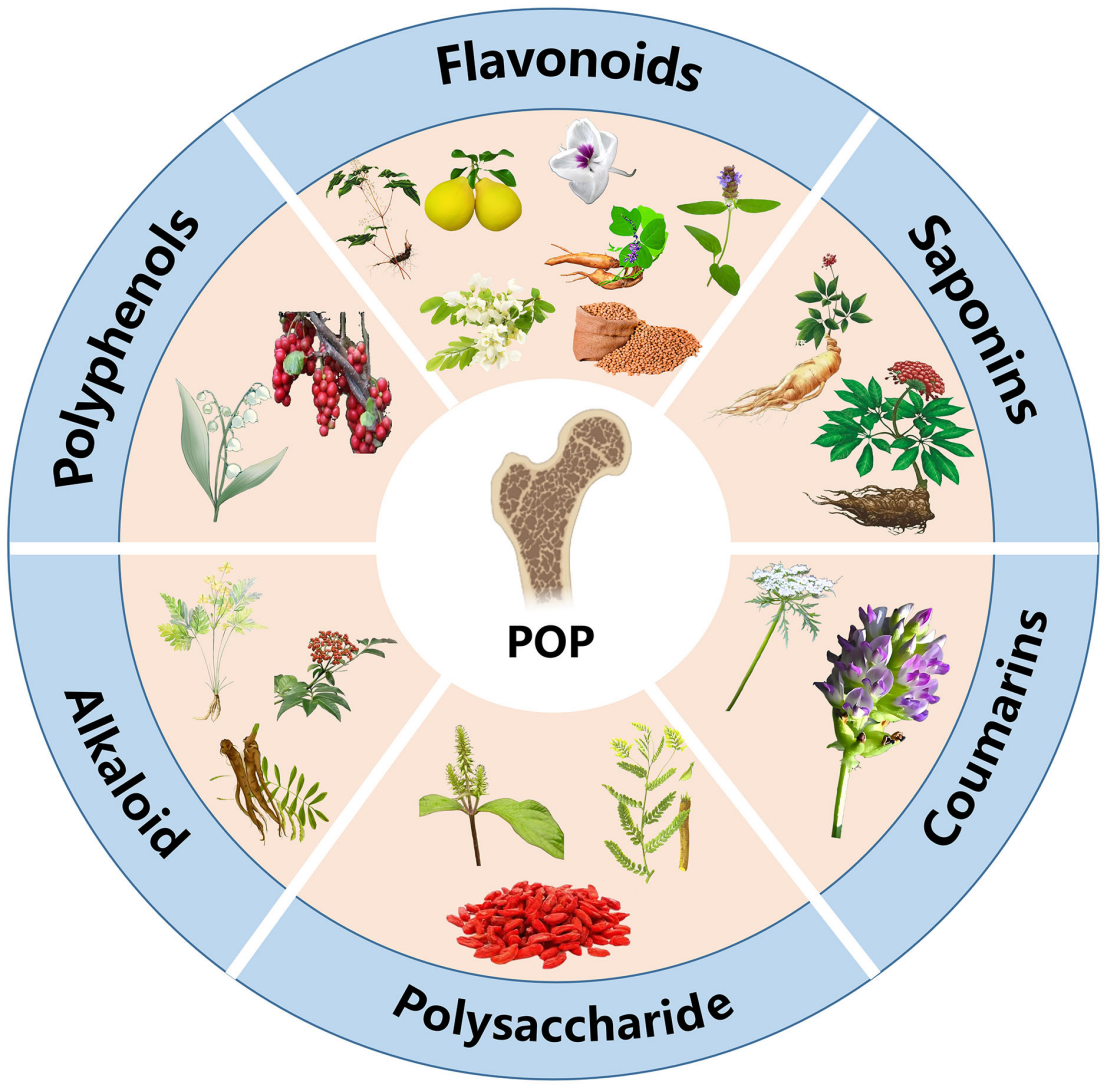
Active constituents of traditional Chinese medicine for the treatment of POP.

Consequently, identifying safer and more efficacious treatments is pivotal to the management of POP. The active constituents of TCM modulate bone metabolism via multifaceted, multi-target pathways, and certain chemicals can be used in combination with other drugs to enhance their effectiveness or alleviate their adverse effects. Panax ginseng saponins, flavonoids, and polysaccharides have shown potential efficacy in synergistically enhancing bone mass in arthritic rats and were well tolerated (16). The active constituents of Chinese medicines compensate for the shortcomings inherent in the intricate composition of traditional compound formulae and the ambiguity surrounding their mechanisms. As one of the key characteristics of TCM therapy, the active constituents in herbs offer diverse therapeutic options for patients and support the health of those with OP. This review delineates the role and application of active constituents in TCM therapy for the treatment of POP, utilizing databases such as China National Knowledge Infrastructure and PubMed to provide a comprehensive overview of diverse treatment options available to patients (**Figure 1**). The objective is to provide novel insights into the prevention and treatment of OP through TCM and its active constituents.

## 2 Pathogenesis of POP

**Figure 2:**
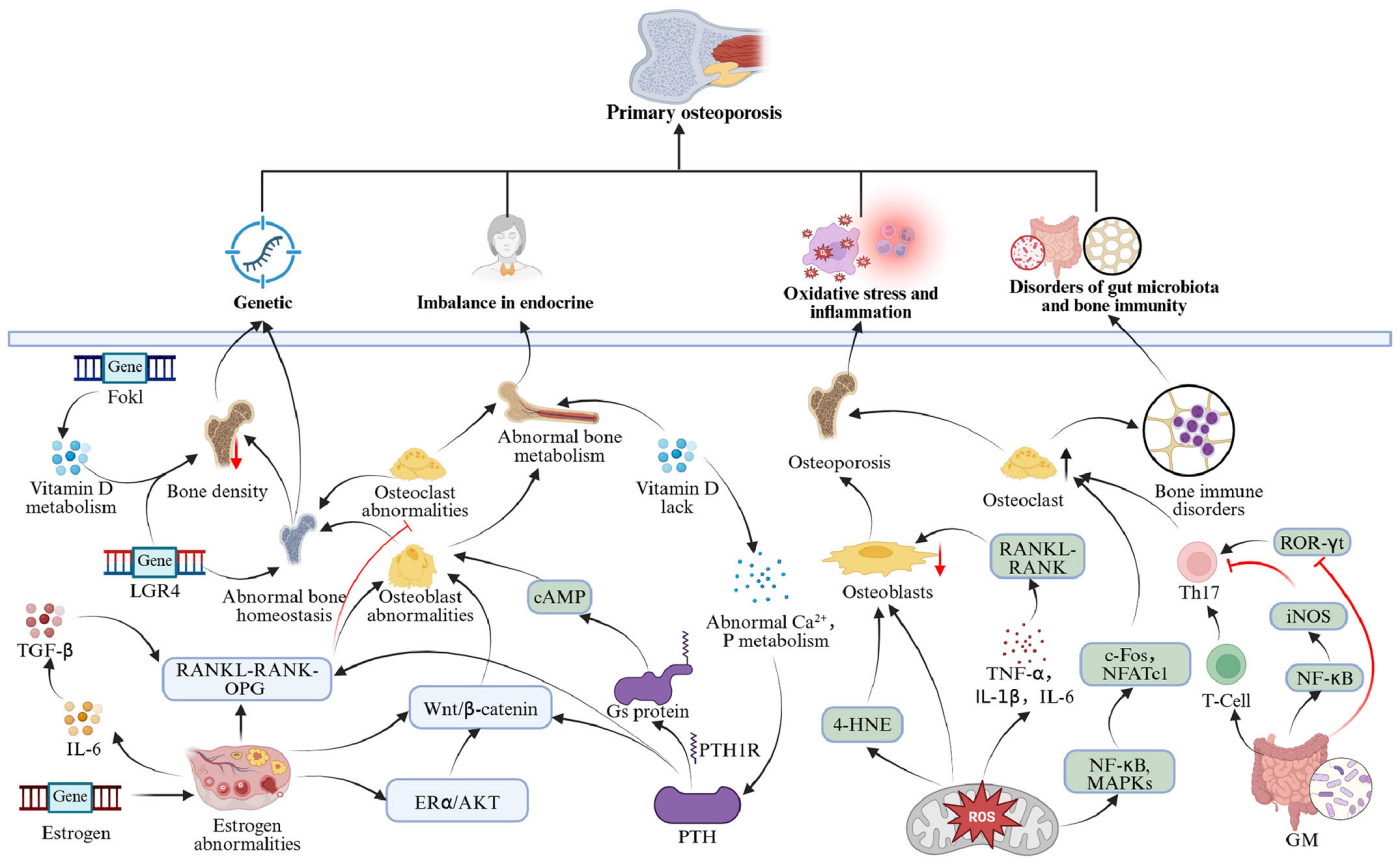
Mechanism diagram of POP.

POP arises from the interplay of genetic, endocrine, microenvironmental, and gut microbiota variables, characterized by a complex pathophysiology involving multi-system and multi-pathway interactions (**Figure 2**).

### 2.1 Hereditary influences

Genetic factors significantly contribute to the etiology of OP. Genome-wide association studies and candidate gene analyses have discovered several significant gene variants strongly linked to diminished bone mineral density (BMD) and increased fracture risk. The genetic influence on OP differs by phenotype. Specifically, hereditary predisposition to osteoporotic fractures is around 25%, wrist fractures range from 25% to 54%, and hip fractures can reach up to 48% (17). Genome-wide association studies systematically revealed the molecular network of OP susceptibility genes, mainly involving the following pathways: vitamin D metabolism pathways (VDR, DBP), estrogen signaling (ESR1, ESR2, CYP19A1), the Wnt/β-catenin pathway (LRP5, SOST, WNT10B), and the RANKL-RANK-OPG system (TNFRSF11A, TNFRSF11B) (18). Polymorphisms in the vitamin D receptor (VDR) gene are among the most well-investigated genetic determinants. Research involving 174 postmenopausal women (aged 43–71) revealed the following distribution of the FokI genotype: FF (33.3%), Ff (50.6%), and ff (16.1%). The ff genotype exhibited a significantly reduced lumbar spine BMD compared to those with the FF genotype, and the prevalence of the ff genotype was markedly greater in the OP cohort than in the normal bone mass cohort, indicating that FokI polymorphism may affect bone metabolism by modulating VDR protein function (19). Studies have identified several single nucleotide polymorphisms linked to OP, including rs1061947 (COL1A1), rs10793442 (ZNF239), and rs11614913 (miR-196a), which are associated with fracture risk, while rs5854 (MMP1) and rs2910164 (miR-146a) correlate significantly with low BMD. And, rs10098470 (TPD52), rs11540149 (VDR), rs1042673 (SOX9), rs1054204 (SPARC), and rs1712 (FBXO5) have been identified as prevalent genetic markers for fractures and low BMD (20). Family studies have corroborated that the rs11029986 allele of the LGR4 gene is associated with hip BMD, whereas the rs12796247 and rs2219783 polymorphisms affect lumbar spine BMD (21). Key genetic variables associated with POP, particularly in its early stages, include the core genes LRP5, COL1A1, COL1A2, WNT1, and PLS3 (22). A study in the Volga-Ural region of Russia has demonstrated that hypomethylation of the RUNX2 gene’s promoter region is associated with POP, specifically at the CpG1 locus. The CpG1 locus may serve as a potential biomarker, with a more pronounced epigenetic profile observed in male individuals (23). Moreover, miR-422a may facilitate the lipogenic differentiation of human bone marrow mesenchymal stem cells (BMSCs) by downregulating MeCP2, potentially resulting in increased bone marrow adiposity and decreased bone production (24). These genetic factors interact through various pathways to establish the molecular foundation of OP, collectively regulating the balance between osteogenesis and resorption and influencing bone homeostasis.

### 2.2 Endocrine regulatory dysregulation

An imbalance in the endocrine system is a fundamental factor contributing to the start and progression of OP, predominantly characterized by estrogen insufficiency, abnormalities in parathyroid hormone (PTH), and disorders in vitamin D metabolism.

Estrogen irregularities: Estrogen is integral to bone metabolism via its receptors (ERα and ERβ), sustaining metabolic equilibrium in bone, and is a fundamental pathway in POP (25). Postmenopausal estrogen insufficiency results in an altered RANKL/OPG ratio, increased osteoclast activation, and expedited bone resorption. Estrogen specifically facilitates the production of OPG and suppresses RANKL-induced osteoclast differentiation (26). Moreover, estrogen diminishes the secretion of bone resorption factors such as IL-6 and RANKL while augmenting the activity of bone formation factors like TGF-β (27). At the molecular level, estrogen activates the ERα/Akt signaling pathway, which subsequently enhances the activation of the Wnt/β-catenin signaling pathway, thereby stimulating osteoblast proliferation and differentiation (28, 29).

Elevated PTH: PTH is crucial for regulating calcium metabolism, and older individuals with OP frequently exhibit increased PTH levels. This may result from age-related renal impairment, which diminishes the production of 1,25(OH)_2_D_3_, thereby reducing intestinal calcium absorption, lowering serum calcium levels, and subsequently inducing elevated PTH secretion, which promotes bone resorption (30). PTH interacts with the PTH1 receptor (PTH1R) to initiate the G protein-coupled signaling cascade, consequently modulating two principal pathways: protein kinase A (PKA) and protein kinase C. Upon binding to PTH1R, PTH activates the Gs protein, which stimulates adenylate cyclase to produce cAMP, thereby activating PKA and controlling the differentiation, proliferation, and death of osteoblasts. PTH can activate phospholipase C via Gq protein, producing IP3 and DAG, the latter of which activates protein kinase C, thus affecting the expression of genes associated with bone metabolism (31, 32).

Disorders in vitamin D metabolism: Inadequate levels of active vitamin D can result in disturbances in calcium and phosphorus metabolism, hence impacting bone mineralization (33). Vitamin D deficiency diminishes intestinal calcium absorption, resulting in decreased blood calcium levels that trigger increased PTH secretion (34). Elevated PTH enhances osteoclast activation by upregulating RANKL expression and concurrently inhibits the Wnt/β-catenin signaling pathway in osteoblasts, culminating in augmented bone resorption and diminished bone formation (35). Clinical studies indicate a significant correlation between vitamin D deficiency and elevated levels of bone metabolism markers, such as PINP, B-CTX, and N-MID (36).

### 2.3 Oxidative stress and inflammatory response

Oxidative stress and chronic inflammation are significant contributors to OP. The excessive accumulation of reactive oxygen species (ROS) disturbs the osteogenic-osteoclastic equilibrium, resulting in heightened osteoclast formation and suppressed osteoblast activity (37). In osteoblasts, oxidative stress impedes the nuclear translocation of nuclear factor E2-related factor 2 (Nrf2), which contributes to increased osteoclast formation (38). The inactivation of Nrf2 leads to the accumulation of ROS in osteoblasts, inducing ferroptosis, characterized by abnormal mitochondrial morphology and elevated lipid peroxidation products (e.g., 4-HNE), which subsequently impedes osteogenic differentiation and results in bone loss (23). Furthermore, ROS activate the NF-κB and MAPK signaling pathways, enhancing the expression of essential osteoclast transcription factors (including c-Fos and NFATc1), therefore facilitating bone resorption (39).

In a chronic low-grade inflammatory condition, pro-inflammatory mediators such as TNF-α, IL-1β, and IL-6 are consistently elevated, promoting osteoclast development through the activation of the RANKL-RANK signaling pathway. Clinical studies indicate that systemic immune inflammation indices are markedly elevated in patients with OP, and when these indices surpass 613.03, the risk of OP significantly escalates (40). Furthermore, monocytes derived from female OP patients can autonomously differentiate into osteoclasts *in vitro* without external stimulation (41), while macrophages and monocytes further facilitate osteoclastogenesis by secreting IL-1 and TNF-α (42).

### 2.4 Gut microbiota and bone immune dysfunction

The gut microbiota modulates bone metabolism via the “gut-bone axis,” employing mechanisms that include immune modulation and metabolite-mediated signaling pathways (43). Research indicates that gut microbiota can affect the equilibrium of Th17 and Treg cells by modulating the differentiation and functionality of immune cells. Th17 cells secrete pro-inflammatory mediators, including IL-17, RANKL, and TNF-α, which directly facilitate osteoclast differentiation and activation while concurrently inhibiting osteoblast activity; conversely, Treg cells produce anti-inflammatory mediators, such as IL-10 and TGF-β, which not only suppress osteoclast formation but also enhance the expression of osteoblast-related factors (44). The disruption of this equilibrium results in bone remodeling disorders, and the overactivation of Th17 cells markedly increases bone resorption via the RANKL/RANK pathway (45). Furthermore, gut microbiota can affect bone remodeling by modulating immune cell function through metabolic byproducts such as short-chain fatty acids (SCFAs) (46). Butyrate can diminish the expression of iNOS, TNF-α, and IL-6 by reducing NFκB transcriptional activity while simultaneously boosting IL-10 expression and suppressing the development of Th17 cells (47, 48). Propionate inhibits histone deacetylase, reducing ROR-γt expression, thereby inhibiting Th17 cell differentiation (49). SUN et al. discovered that the administration of Jiangu granules could modulate intestinal flora homeostasis, enhance the secretion of short-chain fatty acids, adjust the Treg/Th17 cell ratio, and modify the expression of cytokines associated with bone immunomodulation in ovariectomized rats, thereby inhibiting osteoclast differentiation and effectively preventing bone loss (50). Blautia, Parabacteroides, and Ruminococcaceae exhibited notable disparities between osteoporotic and healthy persons, influencing bone health (51). In conclusion, the imbalance of intestinal flora and bone immunological diseases is significantly linked to OP, offering a theoretical foundation for targeting intestinal flora as an intervention strategy.

## 3 Mechanisms and applications of active constituents in the prevention and treatment of POP

### 3.1 Flavonoids

#### 3.1.1 Icariin

**Figure 3:**
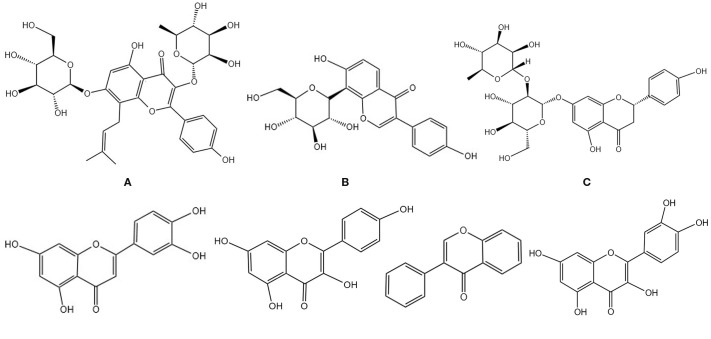
Chemical structural formula of several flavonoids: **(A)** Icariin. **(B)** Puerarin. **(C)** Naringin. **(D)** Luteolin. **(E)** Kaempferol. **(F)** Isoflavone. **(G)** Quercetin.

Icariin is the primary bioactive component of Epimedium brevicornu Maxim, a plant belonging to the Berberidaceae family (**Figure 3A**). In TCM, it is extensively utilized for the management of OP owing to its capabilities in warming and tonifying kidney yang, as well as fortifying tendons and bones. Icariin is the predominant flavonoid glycoside in Epimedium brevicornu Maxim, demonstrating functions that enhance cardiovascular function, bolster immune system efficacy, and regulate the endocrine system, and it exhibits antitumor, antiviral, antihypoxia and reperfusion injury properties (52). Contemporary research demonstrates that icariin treats OP primarily by modulating the levels and functions of osteoclasts and osteoblasts, regulating the differentiation of BMSCs, thereby promoting bone formation, inhibiting bone resorption, and restoring bone mass homeostasis, thus treating OP (53). Regarding the inhibition of bone resorption, icariin can influence the differentiation and function of osteoclasts through various mechanisms. Zhiwei Li et al. discovered that icariin inhibits the formation of the osteoclast cytoskeletal filamentous actin (F-actin) ring in a dose-dependent manner by upregulating the expression of the negative regulatory factor guanine nucleotide-binding protein subunit α13 (Gα13), thereby obstructing the downstream PKB/GSK-3β/NFATc1 signaling pathway and consequently inhibiting osteoclast formation (54). Additionally, Yuhao Si et al. demonstrated that icariin activates the Cullin 3/Nrf2/HO-1 pathway, which inhibits osteoclast differentiation, significantly reduces oxidative stress levels, decreases the number of TRAP-positive osteoclasts in the bone tissue of ovariectomized (OVX) rats, lowers bone tissue ROS levels, and increases BMD (55). Regarding the promotion of bone formation, icariin enhances bone metabolism by regulating the bone marrow microenvironment and stem cell differentiation. Long Bai et al. discovered that icariin stimulates the autophagy process, mitigates the inflammatory aging phenotype of senescent macrophages, and diminishes the release of the senescence-associated secretory phenotype, thereby enhancing the bone immune microenvironment and restoring the osteogenic potential of aged BMSCs, ultimately alleviating OP (52). Shaozi Lin et al. also found that icariin inhibits the Hippo-YAP/TAZ signaling pathway, reduces YAP/TAZ phosphorylation, and suppresses the adipogenic regulator PPARγ, thereby promoting the osteogenic differentiation of ADSCs and inhibiting adipogenic differentiation (56). Therefore, icariin regulates the “bone formation-bone resorption” balance in a bidirectional manner, inhibiting osteoclast-mediated bone resorption while promoting osteogenic differentiation and improving the bone microenvironment, thereby restoring bone mass homeostasis.

#### 3.1.2 Puerarin

Puerarin is an isoflavone compound isolated from the dried rhizomes of wild kudzu [Pueraria montana (Lour.) Merr.] (**Figure 3B**). It possesses anti-inflammatory, antioxidant, and estrogen-like pharmacological effects and can positively influence bone metabolism by modulating multiple signaling pathways (57). Research indicates that puerarin modulates osteoclast differentiation by obstructing the TRAF6/ROS-dependent MAPK/NF-κB signaling pathway. This is achieved through the downregulation of NOX1 expression, reduction of ROS production, and upregulation of HO-1 levels, which collectively inhibit the activation of the MAPK and NF-κB pathways, leading to the downregulation of osteoclast-specific genes such as NFATc1, MMP9, and CTSK (58). Furthermore, it facilitates osteoblast differentiation through the activation of the ERK1/2 and p38-MAPK pathways, markedly enhancing BMD, bone volume fraction (BV/TV), and trabecular number (Tb.N) in OVX rats (59). Additionally, puerarin suppresses the activation of the JAK2/STAT3 signaling pathway, efficiently mitigating bone loss and microstructural damage in postmenopausal osteoporosis(PMOP) rats (60). A meta-analysis established that puerarin exhibits a significant bone-sparing effect on ovariectomy-induced PMOP, demonstrating efficacy comparable to estrogen and enhanced safety (61). These results offer a robust theoretical foundation for the prospective application of puerarin as a therapeutic agent for POP.

#### 3.1.3 Naringin

Naringin is a natural flavonoid compound (**Figure 3C**) and one of the active constituents of the TCM Drynariae Rhizoma. It possesses multiple pharmacological effects, including promoting bone growth, anti-inflammatory activity, and promoting microvascular regeneration (62). The mechanism of action of naringin in regulating POP has been widely studied, and the existing evidence shows that it can regulate POP activity through multiple pathways. Yubo Cui et al. found that naringin promotes osteoblast differentiation by activating the Wnt/β-catenin signaling pathway, inhibiting bone resorption and thereby improving OP in OVX mice (63). Hui Wang et al. found that naringin binds to ESR1 and HSP90AA1, activates the Wnt/β-catenin and PI3K/Akt signaling pathways, induces GSK-3β phosphorylation, promotes β-catenin nuclear translocation, thereby reversing the inhibitory effects of oxidative stress on osteoblast differentiation (64). Naringin may influence OPG mRNA expression in a time- and dose-dependent manner, facilitating the secretion of OPG protein, upregulating OPG expression, and increasing the OPG/RANKL ratio, which inhibits osteoclast differentiation and activation, thereby diminishing bone resorption, concurrently, it synergistically enhances OPG secretion with vitamin D_3_, further substantiating its stimulatory effect on bone formation (65). Wang Wang et al. demonstrated that naringin inhibits the JAK2/STAT3 signaling pathway, enhances the proliferation and osteoblast differentiation of BMSCs, and diminishes osteoclast activity, thereby rectifying the imbalance between bone formation and resorption in PMOP rats, modulating bone metabolism, and mitigating OP (66). Furthermore, naringin decreases VEC apoptosis by attenuating endoplasmic reticulum stress (downregulating GRP78, CHOP, and caspase-12) and mitochondrial-mediated apoptosis pathways while also regulating the ET/NO balance to facilitate bone vascularization (67). So, naringin regulates osteoblasts to treat OP by modulating signaling pathways, including the Wnt/β-catenin, JAK2/STAT3, PI3K/Akt, and estrogen receptor signaling pathways, as well as mitochondrial apoptosis pathways, thereby regulating osteoblast function and treating OP.

#### 3.1.4 Luteolin

Luteolin is a naturally occurring flavonoid compound (**Figure 3D**) found in medicinal plants such as Dendranthema morifolium, Lonicera japonica Thunb, Prunella vulgaris L., and Eclipta prostrata L. Luteolin is frequently utilized in TCM for “tonifying the kidneys and strengthening bones.” As an active constituent of various medicinal plants, luteolin exhibits substantial therapeutic effects in the prevention and treatment of OP through multifaceted regulatory mechanisms. Research indicates that luteolin not only enhances the osteogenic differentiation of BMSCs by activating the PI3K-Akt signaling pathway (68), mitigates osteoclast pyroptosis and improves bone microstructure, (69) but also inhibits the RANKL signaling pathway to downregulate transcription factors such as NFATc1, thereby synergistically diminishing levels of inflammatory factors like TNF-α and IL-6 (70). Luteolin significantly improves osteoblast function via estrogen receptors and the Wnt/β-catenin pathway (71). Furthermore, in an OVX animal model, it exhibited efficacy akin to estrogen replacement therapy without associated carcinogenic risks (70), underscoring its potential as a safe and effective anti-OP agent.

#### 3.1.5 Kaempferol

Kaempferol is a flavonoid active compound extracted from various plants, exhibiting anti-inflammatory, antitumor, antioxidant, and anti-allergic properties (**Figure 3E**). Kaempferol can prevent and treat OP through various signaling pathways (72). Research indicates that kaempferol enhances BMD in OVX rats and increases the bone mineralization capacity and ALP activity of BMSCs. The underlying mechanism may involve the downregulation of miR-10a-3p, which alleviates its post-transcriptional inhibition of CXCL12, consequently upregulating CXCL12 expression, promoting BMSC osteogenic differentiation, and ameliorating ovariectomy-induced OP in rats (73). YAP is a key transcriptional cofactor in the Hippo signaling pathway, which promotes osteoblast differentiation (74). The NF-κB signaling pathway activation is associated with inflammation, promoting osteoclast differentiation and inhibiting osteogenesis (75). Wencheng Liu et al. found that kaempferol alleviates the inhibition of YAP via the Hippo pathway, promotes YAP nuclear translocation, thereby upregulating the expression of osteogenesis-related genes, and inhibits RANKL-mediated osteoclast differentiation. Additionally, YAP inhibits the phosphorylation and nuclear translocation of NF-κB-p65 by binding to its subunits, reducing the release of inflammatory factors, thereby weakening osteoclast differentiation signals (76).

#### 3.1.6 Soy isoflavones

Soy isoflavones is a natural phytoestrogen belonging to the flavonoid compound family and is widely present in leguminous plants, where it is an important secondary metabolite formed during growth (**Figure 3F**). Studies have shown that the primary active metabolite of soy isoflavone, equol, binds to ERβ receptors, upregulates the OPG/RANKL pathway, and promotes osteoblast proliferation while inhibiting apoptosis in a dose-dependent manner, thereby exerting a protective effect against PMOP (77). Bing et al. further confirmed that the soy isoflavone metabolite equol can promote osteoblast secretion of OPG through ERβ, significantly increase the OPG/RANKL ratio, and effectively inhibit the activation of the RANKL/RANK signaling pathway, thereby suppressing osteoclast activation (78). Additionally, soy isoflavones can promote the differentiation of BMSCs into osteoblasts by activating the Wnt/β-catenin signaling pathway (79). Animal experiments have confirmed that a dose of 60 mg/kg of soy isoflavone can bidirectionally regulate the activity of osteoblasts and osteoclasts, with its effects on improving BMD and bone microstructure comparable to those of estrogen therapy (80). These findings provide robust experimental evidence supporting soy isoflavone as a safer alternative to estrogen therapy.

#### 3.1.7 Quercetin

Quercetin (**Figure 3G**) is a flavonol substance exhibiting numerous biological actions, predominantly located in the stem bark, flowers, leaves, buds, seeds, and fruits of various plants, frequently as glycosides. Quercetin exhibits anti-osteoporotic effects through various synergistic mechanisms, including the promotion of bone formation, inhibition of bone resorption, anti-inflammatory properties, and antioxidative stress, thereby providing a theoretical foundation for the development of innovative OP therapeutic strategies (81). Quercetin enhances BMD, improves bone microstructure and biomechanical properties, inhibits bone resorption, promotes bone formation, enhances muscle morphology and locomotion, and decreases fracture risk in denuded mice by modulating the GPRC6A/AMPK/MTOR-mediated glycolipid metabolism pathway (82). Ruibing Feng et al. discovered that quercetin can modulate intestinal microbiota, enhance the synthesis of SCFAs, suppress inflammation, and diminish the risk of OP. SCFAs modulate the “gut flora - SCFAs - inflammation” axis to ameliorate OP and suppress inflammatory responses, hence safeguarding bone integrity in ovariectomized rats (83). A study utilizing *in vitro* cellular experiments and *in vivo* animal models revealed that quercetin enhances iron and vitamin B2/HO-1 signaling pathways by activating the Nrf2/HO-1 signaling pathway, which mitigates iron overload-induced apoptosis and oxidative stress, thereby restoring osteoblast function and alleviating OP (84).

### 3.2 Polyphenolic

#### 3.2.1 Resveratrol

**Figure 4:**
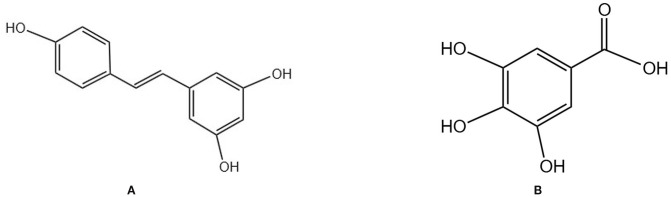
Chemical structural formula of several polyphenols: **(A)** Resveratrol. **(B)** Gallic acid.

Resveratrol is a natural plant-derived polyphenolic compound (**Figure 4A**) with antioxidant, anti-inflammatory, anti-aging, and phytohormonal activities. Resveratrol is found in various TCM plants, including Mori Fructus and Giant knotweed rhizome (85). It operates through multiple mechanisms, such as modulating gut microbiota, enhancing intestinal barrier integrity, and facilitating epigenetic regulation (86). A 24-month randomized, double-blind, placebo-controlled, crossover trial indicated that long-term resveratrol supplementation (75 mg bid) significantly enhanced BMD in the lumbar spine and femoral neck of postmenopausal women, decreased bone resorption, and may correlate with phytoestrogen effects, improved systemic vascular function, and synergistic interactions with vitamin D/calcium (87). A preclinical study further demonstrated that resveratrol can markedly enhance BMD and microstructure in animal models of POP by modulating bone metabolism signals, exhibiting antioxidant and anti-inflammatory properties, and displaying estrogen-like activity. The dosage range is specified as 5–200 mg/kg/day, with more significant effects observed at doses of 40–80 mg/kg/day (88). An animal study corroborated that resveratrol markedly enhanced BMD in OVX rats, with the underlying mechanism involving the upregulation of the Wnt/β-Catenin signaling pathway, which promotes osteogenesis and inhibits osteoclast activity, thus rectifying bone metabolic imbalance (89).

#### 3.2.2 Gallic acid

Gallic acid is a natural polyphenol (**Figure 4B**) primarily derived from TCMs, such as Galla Chinensis, and exhibits anti-inflammatory and antioxidant properties. It demonstrates clear anti-bone loss effects both *in vitro* and *in vivo*. Peng Zhang et al. found that GA improves BMD, BV/TV, trabecular thickness, and trabecular number in OVX mice while reducing trabecular spacing. It also reduced fat accumulation and osteoclast numbers in bone tissue of OVX mice by inhibiting the Akt, ERK, and JNK signaling pathways and downregulating the NFATc1/c-Fos/CTSK axis, thereby suppressing osteoclast generation (90).

### 3.3 Saponins

#### 3.3.1 Ginsenosides

**Figure 5:**
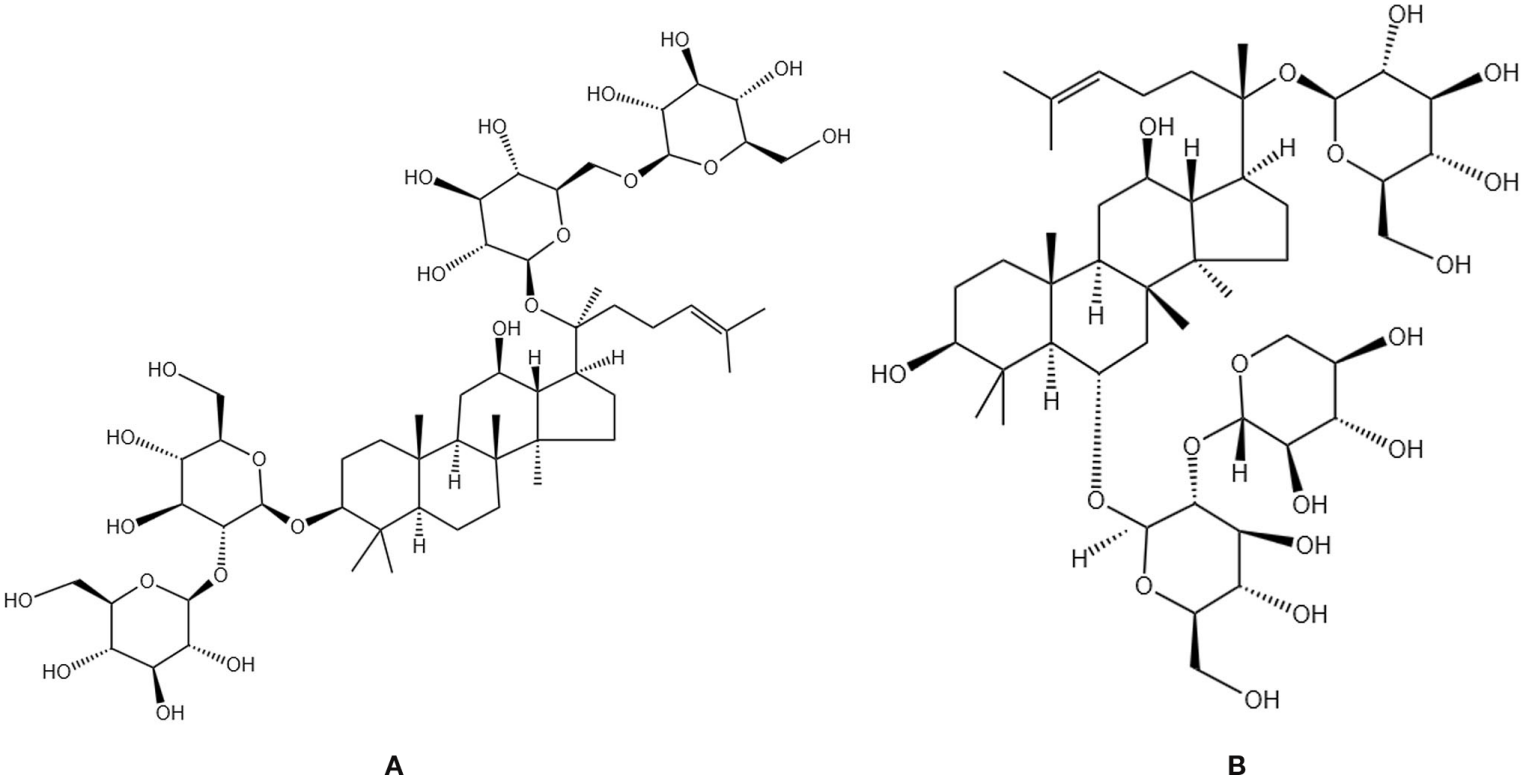
Chemical structural formula of several saponins: **(A)** Ginsenoside Rb 1. **(B)** Notoginsenoside R1.

Ginsenosides are the primary active constituents of Ginseng Radix et Rhizoma (**Figure 5A**), which are essentially non-toxic to normal human cells. Ginsenosides can elevate the expression of critical markers, including Runx2 and ALP, in osteoblasts, thereby promoting mineralization while simultaneously diminishing osteoclast generation and activity. This results in a reduction of TRAP (+) multinucleated cells and RANKL levels, an increase in OPG expression, and antioxidant effects through enhanced glutathione levels and decreased production of ROS and nitric oxide (91). Research indicates that ginsenosides with distinct structures influence biological processes via specific molecular mechanisms. Fei Xi et al. observed an increase in bone mass and enhancement of bone metabolic markers in OVX rats administered ginsenoside Rg3, potentially linked to the modulation of the RANKL/RANK/TRAF6 signaling pathway, which regulates bone metabolism and osteoclast activity in PMOP (92). Shanfu Wang et al. discovered through animal and cellular experiments that ginsenoside Rc elevates the levels of TGF-β, BMP2, and p-Smad2/3 proteins in OVX rats, activates the TGF-β/Smad pathway and concurrently enhances the mRNA and protein expression of Col1a1 and Col1a2, as well as increases alkaline phosphatase activity, indicating promotion of collagen synthesis and bone matrix formation, thus ameliorating OP symptoms in OVX rats (93). Ginsenoside Rg2 inhibits the phosphorylation of the MAPK pathway, downregulates the expression of c-Fos and NFATc1, and consequently diminishes the transcription of osteoclast-specific markers (Acp5, Oscar), establishing a regulatory axis of “MAPK-c-Fos/NFATc1-osteoclast markers,” thereby significantly impeding RANKL-induced osteoclast differentiation (94). Ginsenoside Rh2 may modulate the OPG/RANKL signaling pathway to inhibit osteoclast differentiation and activity, while enhancing Runx2 expression, thereby facilitating osteoblast development and bone formation. This results in increased bone mass and density in aged rats, as well as improved bone microstructure and strength (95). Clinical studies have corroborated that ginsenoside extract administered at a dosage of 3 g/day for 12 weeks significantly elevated serum osteocalcin levels and decreased the DPD/OC ratio in postmenopausal women, demonstrating favorable safety (96). These mechanisms collectively form the molecular foundation for the multifaceted enhancement of bone metabolism by ginsenosides, which is achieved through their antioxidant properties, promotion of osteogenic differentiation, and inhibition of osteoclast activity.

#### 3.3.2 Notoginsenosides

Notoginsenosides are the primary active constituents of the traditional Chinese medicinal herb Notoginseng Radix (**Figure 5B**), derived from the dried roots and rhizomes of the Araliaceae family plant Panax notoginseng. Notoginsenosides R1 can augment BMD and osteoblast activity, as well as facilitate fracture healing (97). Cellular experiments have demonstrated that notoginsenosides R1 mitigates oxidative stress-induced mitochondrial damage by inhibiting the JNK signaling pathway, decreases cell apoptosis, and fosters osteogenic differentiation (98). Ting Wang et al. discovered that notoginsenosides R1 enhances bone formation by activating ERα/β receptors, stimulating ERE-mediated transcription, upregulating osteogenic gene expression, modulating the OPG/RANKL ratio, inhibiting bone resorption, thus facilitating bone formation, while also diminishing reproductive toxicity (99). Yi Liu et al. found that notoginsenosides R1 may promote the proliferation, differentiation, and mineralization of pre-osteoblasts MC3T3-E1 by activating the p38 MAPK or Wnt signaling pathways, regulating transcription factors such as Runx2 and Osterix and significantly enhancing bone formation (100).In OVX rat experiments, total ginsenosides from Panax notoginseng can improve bone tissue damage by upregulating CTRP6 expression, inhibiting RhoA/Rock pathway activation, and alleviating inflammatory responses and oxidative stress (101). These findings systematically elucidate the multifaceted mechanisms by which notoginsenosides improve bone metabolism through “antioxidant-promoting osteogenesis-inhibiting osteoclasts”.

### 3.4 Polysaccharides

#### 3.4.1 Lycium barbarum polysaccharide

Lycium barbarum polysaccharide is the main active ingredient, exhibiting antioxidant, anti-ageing, immune-modulating, and anti-OP effects (102). Studies have shown that lycium barbarum polysaccharide not only activates the Wnt/β-catenin signaling pathway to upregulate the expression of β-catenin and Wnt10b proteins (103), promoting the differentiation of BMSCs into osteoblasts but also by regulating gut microbiota structure (promoting Lactobacillus proliferation) to increase short-chain fatty acid (acetic acid, propionic acid, butyric acid) production (104), thereby promoting bone matrix mineralization through the BMP-2/RUNX2 pathway (105). In an OVX rat model, lycium barbarum polysaccharide improves bone remodeling metabolism by upregulating serum TGF-β1 and NOS levels while alleviating oxidative stress-induced damage to osteoblasts by enhancing SOD and GSH-Px activity (106). These findings systematically reveal that lycium barbarum polysaccharide improve bone metabolism through a triple action mechanism of “directly promoting bone formation, indirectly regulating the microbiota, and synergistically exerting antioxidant effects”.

#### 3.4.2 Astragalus polysaccharide

Astragalus polysaccharide is the primary macromolecular active component of the Astragali Radix, exhibiting various biological functions, including anti-inflammatory, antioxidant, immunomodulatory, and osteogenic effects (107). Extensive studies have confirmed that astragalus polysaccharide can prevent and treat OP by promoting osteogenesis and inhibiting osteoclast activity. In animal models, astragalus polysaccharide significantly improved OP in OVX rats by regulating the FoxO3a/Wnt2/β-catenin signaling pathway, with mechanisms including inhibiting FoxO3a mRNA expression while activating the transcription of Wnt2, LRP5, and β-catenin, thereby increasing BMD, optimizing bone biomechanical properties, and reducing fracture risk (108). At the cellular level, astragalus polysaccharide promotes the proliferation and osteogenic differentiation of human BMSCs in a concentration-dependent manner (optimal concentration: 200 μg/ml),a mechanism potentially involving the inhibition of miR-760 expression and the relief of its transcriptional repression on ANKFY1 (109). Additionally, astragalus polysaccharide can effectively alleviate iron overload-induced functional impairment in BMSCs by inhibiting mitochondrial ROS accumulation, maintaining cell proliferation capacity, suppressing apoptosis and senescence, and preserving pluripotency gene expression (110). Furthermore, astragalus polysaccharide can also promote osteogenesis-related gene expression by activating the BMP-2/Smads signaling pathway through upregulating the expression of BMP-2, p-Smad1, and p-Smad5, thereby improving bone microstructure and bone metabolic indicators in OVX rats (111).

#### 3.4.3 Achyranthes bidentata polysaccharide

Radix Achyranthis Bidentatae, a TCM used to tonify the liver and kidneys and strengthen the tendons and bones, contains the active component achyranthes bidentata polysaccharide (ABP), which exhibits multi-target regulatory effects in the prevention and treatment of OP. Yang Hao et al. found that it can activate the Wnt/β-catenin pathway, promote the expression of genes and proteins such as β-catenin, Runx2, and Osterix, significantly improve bone metabolism in osteoporotic fracture rats, increase BMD, and alleviate bone tissue pathological damage (112). Dezhi Song et al. found that bone marrow mononuclear cells and bone marrow macrophages, 10 μM ABP can inhibit RANKL-induced MAPK phosphorylation and c-Fos expression, thereby blocking the activation of the NFATc1 signaling pathway, and achieving a full-cycle inhibition of osteoclast differentiation, fusion, and bone resorption (113). Additionally, the soluble polysaccharide ABPB and its purified component ABPB-3, from A,chyranthes bidentata exhibit anti-osanti-osteoporotics by improving bone microstructure and increasing bone matrix synthesis (114). The mechanism of ABP also involves regulating the OPG/RANKL/RANK system by upregulating OPG and downregulating RANKL expression to inhibit osteoclast activity while increasing bone formation markers (OC, BAP) levels and reducing bone resorption markers (TPACP5b, NTX, CTX) levels, thereby improving bone metabolism and enhancing bone biomechanical properties in elderly osteoporotic rats (115). These findings collectively reveal the multidimensional pharmacological effects of ABP in preventing and treating OP through a bidirectional regulatory mechanism of “promoting bone formation and inhibiting bone resorption”.

### 3.5 Coumarin derivatives

#### 3.5.1 Osthole

**Figure 6:**
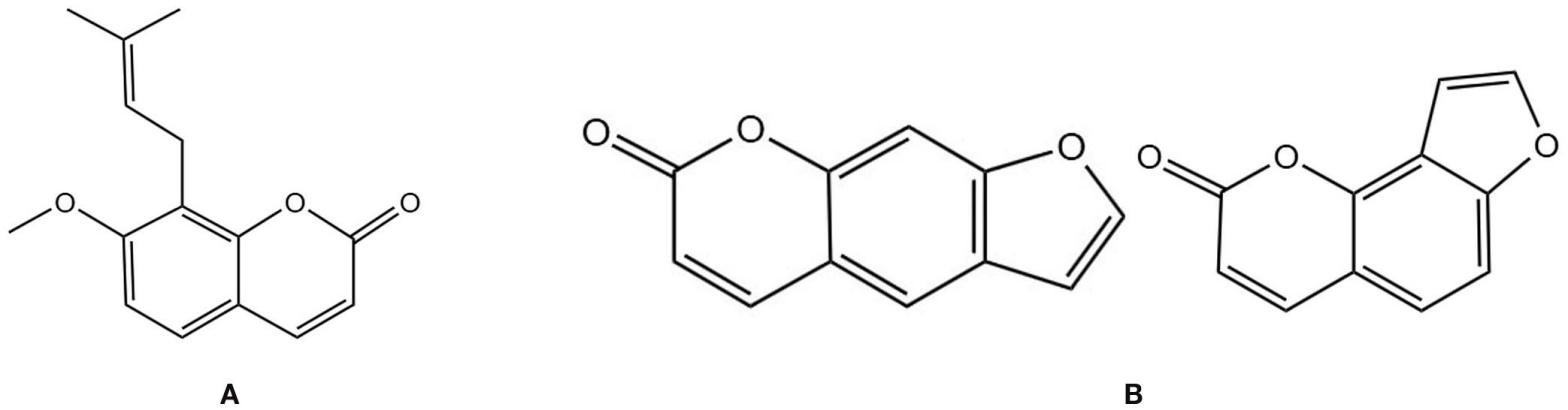
Chemical structural formula of several coumarins: **(A)** Osthole. **(B)** Psoralen and Isopsoralen.

Osthole is a coumarin compound from Cnidii Fructus (**Figure 6A**). It promotes osteoblast differentiation and bone formation while inhibiting bone resorption (116). Studies have shown that osthole significantly increases bone mass in aged mice while inhibiting osteoclast formation and promoting OPG expression. This mechanism may involve activating the β-catenin-OPG signaling pathway to enhance OPG expression. OPG, as a decoy receptor for RANKL, inhibits the binding of RANKL to RANK, thereby suppressing osteoclast differentiation and activity and reducing bone resorption (117). Sheng Zheng et al. also found that osthole activates the Wnt/β-catenin pathway, inducing osteogenic-angiogenic coupling in BMSCs, upregulating the mRNA and protein expression of osteogenic markers (ALP, OCN) and angiogenic factors (VEGFA, CD31), accelerating the healing of tibial fractures in OVX rats, and finding that the optimal dose of osthole was 10 μM (118). Studies have shown that osthole activates key signaling pathways, such as the Wnt/β-catenin and BMP-2/p38 pathways, and upregulates the expression of autophagy-related genes (Beclin1, LC3) in BMMSCs, thereby inducing autophagy. This process promotes the expression of osteogenic-related genes by regulating the activity of transcription factors (such as Runx2 and Osterix) while inhibiting adipogenic differentiation, thereby significantly enhancing the osteogenic differentiation capacity of BMSCs. In an estrogen-deficient OP model, this mechanism was confirmed to effectively increase bone mass and improve bone metabolic indicators (119). Zhong-Rong Zhang et al. found that osthole, on the one hand, upregulates the expression of the transcription factor osterix through the cAMP/CREB signaling pathway, thereby promoting the expression of osteogenesis-related genes such as alkaline phosphatase and osteocalcin; on the other hand, it activates the BMP signaling pathway, producing a synergistic effect with the cAMP/CREB pathway. This dual pathway activation mechanism significantly promotes osteoblast differentiation *in vitro* and exhibits therapeutic effects, including accelerating fracture healing and enhancing bone strength *in vivo (* 120).In terms of metabolomics, Zhenxing Si et al. analyzed and found that osthole can inhibit bone resorption and promote bone formation through 13 metabolic pathways, including linoleic acid metabolism, starch and sucrose metabolism, and arachidonic acid metabolism, thereby effectively improving OP induced by ovariectomy in rats (121).

#### 3.5.2 Psoralen and isopsoralen

Isopsoralen belongs to the furanocoumarin class of extracts from Psoraleae Fructus and is one of the plant estrogens. It is one of the main active constituents of Psoralea corylifolia, a TCM used to tonify the kidneys (**Figure 6B**). It can participate in bone metabolism by regulating the Wnt, Runx2/MMP13, PI3K/AKT, Axin2/PPAR-γ, and WNT/β-catenin signaling pathways, thereby improving OP (122). Studies have shown that psoralen and isopsoralen may regulate the balance of bone remodeling by inhibiting osteoclast activity (reducing TRACP and CTX1) and promoting osteoblast function (increasing ALP), thereby improving bone microstructure and strength in male and female mice with OP induced by sex hormone deficiency (123). Jian Wang et al. found that psoralen can inhibit the expression of PPAR-γ, reducing the differentiation of BMSCs into adipocytes while also alleviating the inhibition of the WNT/β-catenin pathway by PPAR-γ, thereby promoting osteogenic differentiation. Additionally, it can reduce ROS levels, mitigate oxidative stress-induced damage to bone cells, inhibit caspase-3/9-mediated apoptosis, and maintain a balance in bone remodeling, thereby improving OP symptoms in ovariectomized rats (124). Jian Wang et al. also found that isopsoralen can regulate the balance of BMSCs differentiation into osteoblasts by inhibiting PPAR-γ expression and upregulating RUNX2 expression, reducing bone marrow adipogenesis, improving bone mass and microarchitecture in OVX mice without the adverse effects of estrogen replacement therapy, providing a potential option for the prevention and treatment of PMOP (125).

### 3.6 Alkaloids

#### 3.6.1 Berberine

**Figure 7:**
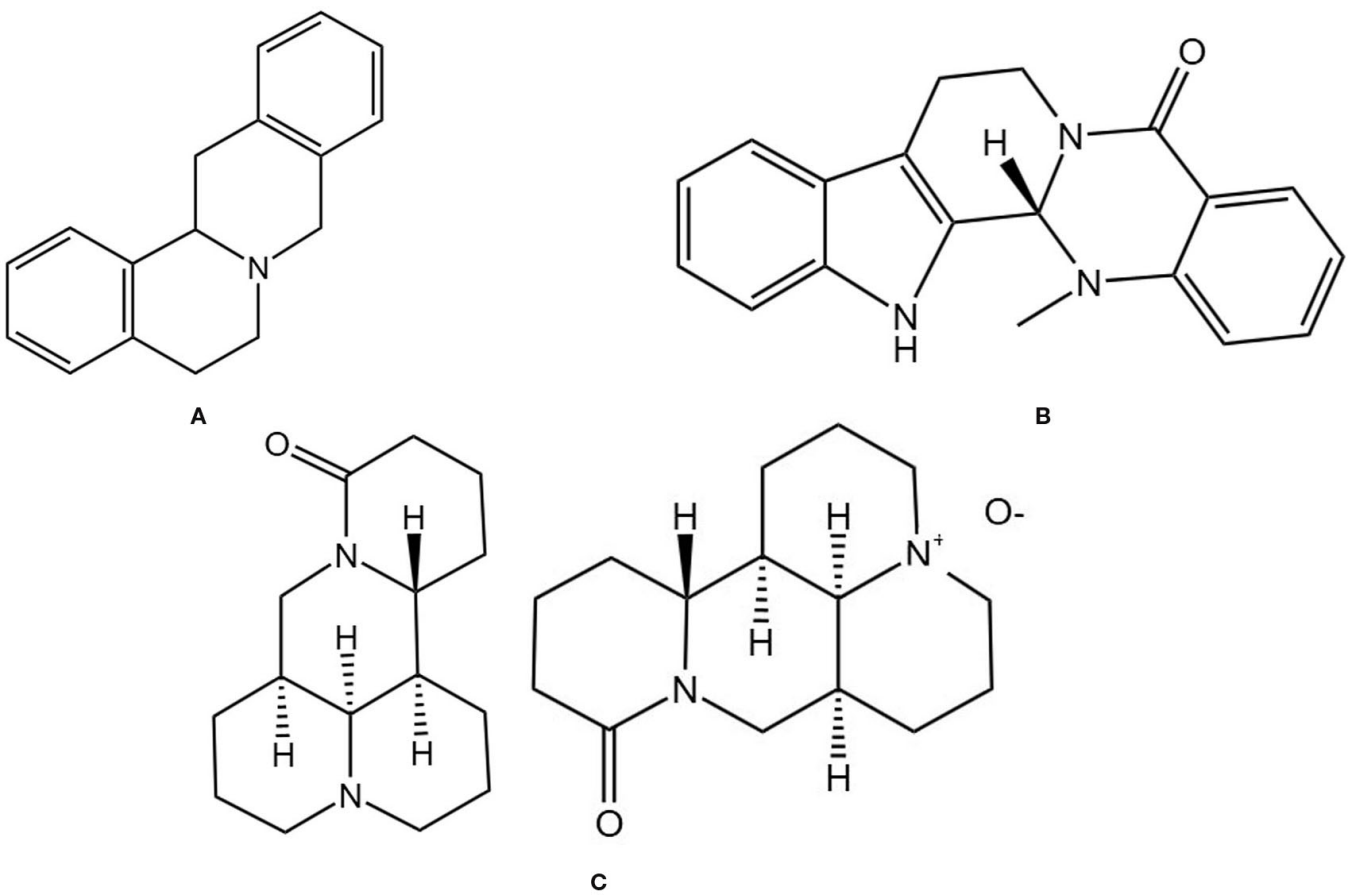
Chemical structural formula of several alkaloid: **(A)** Berbine. **(B)** Evodiamine. **(C)** Matrine and Oxymatrine.

Berberine is an isoquinoline alkaloid (**Figure 7A**) and a major active component of various TCMs. It is widely found in herbs such as Coptis chinensis Franch. and Phellodendri Chinensis Cortex and possesses multiple pharmacological effects, including antidiabetic, antioxidant, and anti-inflammatory activities. In recent years, its multi-mechanistic regulatory role in the prevention and treatment of OP has garnered significant attention. In animal models, berberine activates the cAMP/PKA/CREB pathway to upregulate osteogenic genes, promoting the differentiation of BMSCs into osteocytes. It also downregulates adipogenic genes, inhibiting the differentiation of BMSCs into adipocytes and suppressing osteoclast differentiation. This leads to an increase in trabecular bone volume fraction, number, and thickness in elderly osteoporotic mice while reducing trabecular separation (126). It can also mitigate oxidative stress by inhibiting the RANK/RANKL/OPG pathway, thereby reducing osteoclast activation and alleviating bone mass loss in OVX rats (127). Additionally, berberine can exert bone-protective effects by regulating the “gut-bone axis,” such as enriching butyrate-producing gut microbiota, repairing intestinal barrier integrity, and inhibiting IL-17A-mediated inflammatory responses, thereby alleviating estrogen deficiency-induced periodontal bone resorption (128). These findings suggest that berberine modulates OP through a multidimensional mechanism involving “bone metabolism regulation-oxidative stress inhibition-gut microenvironment improvement”.

#### 3.6.2 Evodiamine

Evodiamine is an alkaloid extracted from the TCM Medcinal Evodia Fruit (**Figure 7B**), exhibiting various bioactivities, including antitumor and anti-inflammatory effects (129). Due to its lipophilic chemical structure and low water solubility, its derivative 3-amino-10-hydroxy evodiamine can inhibit the phosphorylation of NF-κB and MAPK, thereby suppressing the activation of the downstream transcription factor NFATc1 and reducing the expression of genes such as NFATc1, TRAP, CTSK, and DC-STAMP. This inhibits osteoclast differentiation, fusion, and bone resorption functions. This protects bone microarchitecture in OVX mice by reducing bone resorption, promoting osteogenic activity, and maintaining bone mass and structural integrity (130). Haiming Jin also found that evodiamine can inhibit NF-κB-mediated transcription of osteoclast-related genes (such as c-Fos and NFATc1) and inhibit RANKL-induced Ca^2+^ oscillations, thereby blocking NFATc1 activation and its downstream target gene expression, ultimately preventing osteoclast differentiation and maturation, and demonstrating good bone protective effects in OVX mice (131).

#### 3.6.3 Matrine and oxymatrine

Matrine is an alkaloid isolated from the TCM Sophorae Flavescentis Radix (**Figure 7C**). Sophorae Flavescentis Radix is the dried root of the leguminous plant Sophora flavescens (132), which possesses broad pharmacological activities, including anti-inflammatory, anti-fibrotic, and antiviral effects (133). Xiao Chen et al. found that matrine can inhibit RANKL-induced NF-κB, MAPK, and AKT signaling pathways, downregulating NFATc1 and its target genes (MMP-9, TRAP, etc.), thereby inhibiting osteoclast differentiation and bone resorption, improving BV/TV, BMD, and trabecular number in OVX mice, and reducing IL-6, TNF-α, and TRAcp5b levels, effectively preventing bone loss in OVX mice (134). Studies have shown that oxymatrine can increase cortical bone thickness and bone cell numbers in castrated rats, reduce osteoporotic cavities, and exhibit anti-osteoporotic effects comparable to testosterone. The mechanism may involve inhibiting the NF-κB pathway to reduce the release of inflammatory factors and enhancing antioxidant capacity through the Nrf2/HO-1 pathway, thereby regulating the RANKL/OPG balance and inhibiting bone resorption. It may avoid the side effects of testosterone therapy (such as prostate hyperplasia and cardiovascular risks) (135). Further experimental studies are currently needed to investigate the pharmacokinetic characteristics of oxymatrine, providing more data to support its safe and effective use in patients.

## 4 Discussion

This study systematically reviewed the latest research progress in the prevention and treatment of OP using TCM, focusing on the molecular mechanisms by which various active constituents of TCM (including flavonoids, polyphenols, saponins, polysaccharides, coumarins, and alkaloids) regulate bone metabolism through multi-target, multi-pathway synergistic regulation. It has been elaborated on how these active constituents regulate key signaling pathways such as the Wnt/β-catenin, RANKL/OPG pathways while simultaneously intervening in osteoblast differentiation and osteoclast activity, thereby restoring bone metabolic balance. The study particularly emphasizes the unique advantages of TCM constituents in improving the bone microenvironment (such as regulating gut microbiota, inhibiting oxidative stress, and reducing inflammatory responses), as well as their safety profile compared to traditional therapy. By integrating extensive preclinical and clinical research evidence, this study aims to provide a scientific basis for the development of novel anti- OP drugs based on TCM theory and to offer theoretical guidance for the formulation of personalized treatment regimens.

There are also certain limitations at present. (1) Currently, research endorsing the treatment of OP with active compounds from TCM predominantly emphasizes cellular and animal studies, with limited clinical trials in humans. Consequently, it is imperative to enhance the transition from basic to clinical research by employing organoid or 3D bone tissue models to forecast the impact of TCM on the human organism. Additionally, the alterations in biomarkers in patients post-TCM intervention can be examined through metabolomics and proteomics to elucidate its systemic regulatory effects. (2) The bioavailability and formulation optimization of these active substances remain to be addressed, the active constituents of TCM include diverse functions, and when integrated with nanomaterials, they can markedly enhance their bioavailability and bioactivity (136). Numerous studies exist regarding nano-delivery systems and enhanced TCM formulations, including extracellular nanovesicles derived from Herba Epimedium, which can augment the bioavailability and bioactivity of TCM through the PI3K/Akt/mTOR pathway, thereby facilitating the osteogenic differentiation of BMSCs with improved safety (137). And nano-aggregates may demonstrate antipyretic properties by augmenting the solubility of insoluble constituents in Baihu Tang, hence improving cellular absorption and targeted administration (138).Consequently, medication delivery by nanoparticles may emerge as a significant avenue for future research. (3) TCM often employs compound formulas for disease treatment, which are complex and involve multi-component synergistic mechanisms that have not yet been fully elucidated. Consequently, it can be integrated with contemporary analytical techniques, artificial intelligence-assisted design, multi-histology verification, and standardized clinical research to establish a closed-loop of “basic research - mechanism analysis - clinical validation,” or to disassemble the prescription. Alternatively, we can examine the impact of each medicine on OP and subsequently integrate them to confirm the synergistic effect. (4) Due to the variability in each patient’s treatment plan and medication dosage, conducting large-scale western clinical controlled trials is a challenging task. Consequently, we can uphold the principles of TCM diagnosis and treatment by randomly categorizing patients based on the stratification of evidence types, and establishing a structured electronic medical record system to document the information pertaining to the four diagnostic methods of TCM and medication specifics. Subsequently, we can integrate artificial intelligence to analyze medication patterns and standardize the diagnosis and treatment protocols. (5) There is a lack of comprehensive research on the integration of TCM active ingredients with pharmacological techniques, particularly regarding the combination of TCM active ingredients with first-line anti-OP medications. Consequently, subsequent research may concentrate on evaluating combinations of TCM active ingredients for their potential synergistic effects in the prevention and treatment of OP, as well as forecasting the interaction targets and possible effects of TCM active ingredients with western pharmaceuticals, and validating these findings through *in vitro* and *in vivo* experiments.

According to current literature reports, research on TCM treatment for POP is still in the exploratory stage. It is anticipated that future studies will employ new methods and technologies to conduct in-depth investigations, uncover the complex network mechanisms of TCM, and strengthen the integration of basic experimental research with clinical studies, thereby providing a more robust scientific foundation for the clinical application of TCM and the development of new drugs. Future research could also integrate systems pharmacology and artificial intelligence technologies to explore the “component-target-pathway” network of active constituents in TCM, providing a theoretical foundation for the development of novel anti-OP drugs.

The clinical application of active substances in TCM warrants further exploration in the future. In recent years, the integration of active constituents from TCM with exogenous carriers has demonstrated considerable benefits in the prevention and treatment of OP. For example, enteric capsules created by amalgamating epimedium glycosides with snail enzymes markedly enhanced intestinal hydrolysis and absorption efficiency in osteoporotic rats, resulting in a 50% improvement in the total oral bioavailability of epimedium glycosides (139).

In conclusion, investigating the intricate mechanisms of TCM via multidisciplinary technology to enhance clinical efficacy will yield innovative concepts for the advancement of contemporary anti-OP TCM characterized by a “clear mechanism, well-defined ingredients, and stable efficacy”.
